# Electrodiagnostic Confirmation of Lumbar Radiculopathy and Its Association With Lumbar Central Canal Stenosis and Neuroforaminal Stenosis

**DOI:** 10.7759/cureus.69993

**Published:** 2024-09-23

**Authors:** David H Rustom, Arthur Yan, Geoffrey K Seidel

**Affiliations:** 1 Pain Management, Wayne State University Detroit Medical Center, Detroit, USA; 2 Physical Medicine and Rehabilitation, Wayne State University, Detroit, USA; 3 Physical Medicine and Rehabilitation, Wayne State University, Michigan, USA

**Keywords:** chronic low back pain (clbp), emg, lumbar radiculopathy, lumbar spinal stenosis, neuroforaminal stenosis

## Abstract

Introduction: Lumbar spinal stenosis (LSS) and lumbar neuroforaminal stenosis (LNS) are common diagnoses that plague patients with low back pain. Electrodiagnostic testing (EDX) can be used as an adjunct to investigate lower extremity radicular nerve pain and/or neurogenic claudication. However, there are only limited studies discussing the association of these diagnostic tools with radiculopathy. We investigate the association between EDX-confirmed radiculopathy and the degree of LSS and LNS found on MRI.

Methods: A retrospective cohort study of patients presenting to an outpatient pain medicine clinic who had a documented EDX and lumbar MRI. We used a Pearson chi-square test to compare the severity of radiographic LSS/LNS with EDX data. The data were fit to a multivariable logistic regression model.

Results: There were not any statistically significant correlations when comparing EDX evidence of radiculopathy and LSS (p = 0.50), LSS severity (p = 0.54), LNS (p = 0.69), or LNS severity (p = 0.11).

Conclusions: We found no significant associations between LSS/LNS severity and EDX findings. The presence and degree of severity of LSS/LNS on MRI were not reliable predictors of EDX findings.

## Introduction

Lumbar spinal stenosis (LSS) is a degenerative condition that increases with every decade of life, particularly over the age of 65 [1]. The most common treatment modalities for this clinical syndrome include medications, physical therapy, and lumbar epidural steroid injections. Failure of conservative treatment is routinely accompanied by further workup with advanced diagnostic modalities such as magnetic resonance imaging (MRI) and electrodiagnostic studies (EDX). The future challenge, if costs are to be controlled, appears to lie squarely with prevention and optimum management [2].

LSS is used to describe a clinical syndrome associated with back and leg pain. This pain is made worse with prolonged standing or ambulation. Variable degrees of LSS have been described as mild, moderate, and severe. Anatomical narrowing can compress the nerves in the spinal canal and can lead to cauda equina syndrome. Lumbar pain and neurogenic symptoms are frequently relieved with forward flexion [3-5].

The objective of this study was to identify whether or not there is an association between the degree of LSS and EDX studies.

The term LSS refers to a narrowing of the central spinal canal, whereas LNS refers to a narrowing of the neural foramen. Narrowing can be caused by degeneration of the ligamentum flavum, facet joints, or intervertebral discs, leading to an inflammatory response [5,6].

Imaging such as MRI and computed tomography (CT) are utilized as adjuncts for the purpose of visualizing anatomy and pathology in LSS/LNS. These radiographic studies can guide surgical intervention [7]. MRI is the method of choice for assessing the severity of spinal stenosis [7-8]. 

Treatment can be challenging when clinical history, physical examination, and radiographic findings are numerous [7-8]. EDX has been shown to have high specificity for nerve pathology [9-10]. EDX adds prognostic value, which can, in turn, lead to better long-term treatment outcomes, especially for patients with lower extremity radicular pain in the presence of lumbar spinal stenosis [11].

The use of EDX for patients with suspected radiculopathy has been well established [12-15]. EDX results have been shown to be significantly altered in selected patients with moderate to severe degrees of central spinal stenosis [14].

MRI and EDX can often be overutilized in the setting of lumbar radicular pain syndromes [16-18]. Previous studies have shown discrepancies between EDX and MRI results. Notably, differences can exist between structural levels seen on MRI and neurological levels diagnosed on electromyography (EMG) [19].

## Materials and methods

Methods

Patient Selection

**Figure 1:**
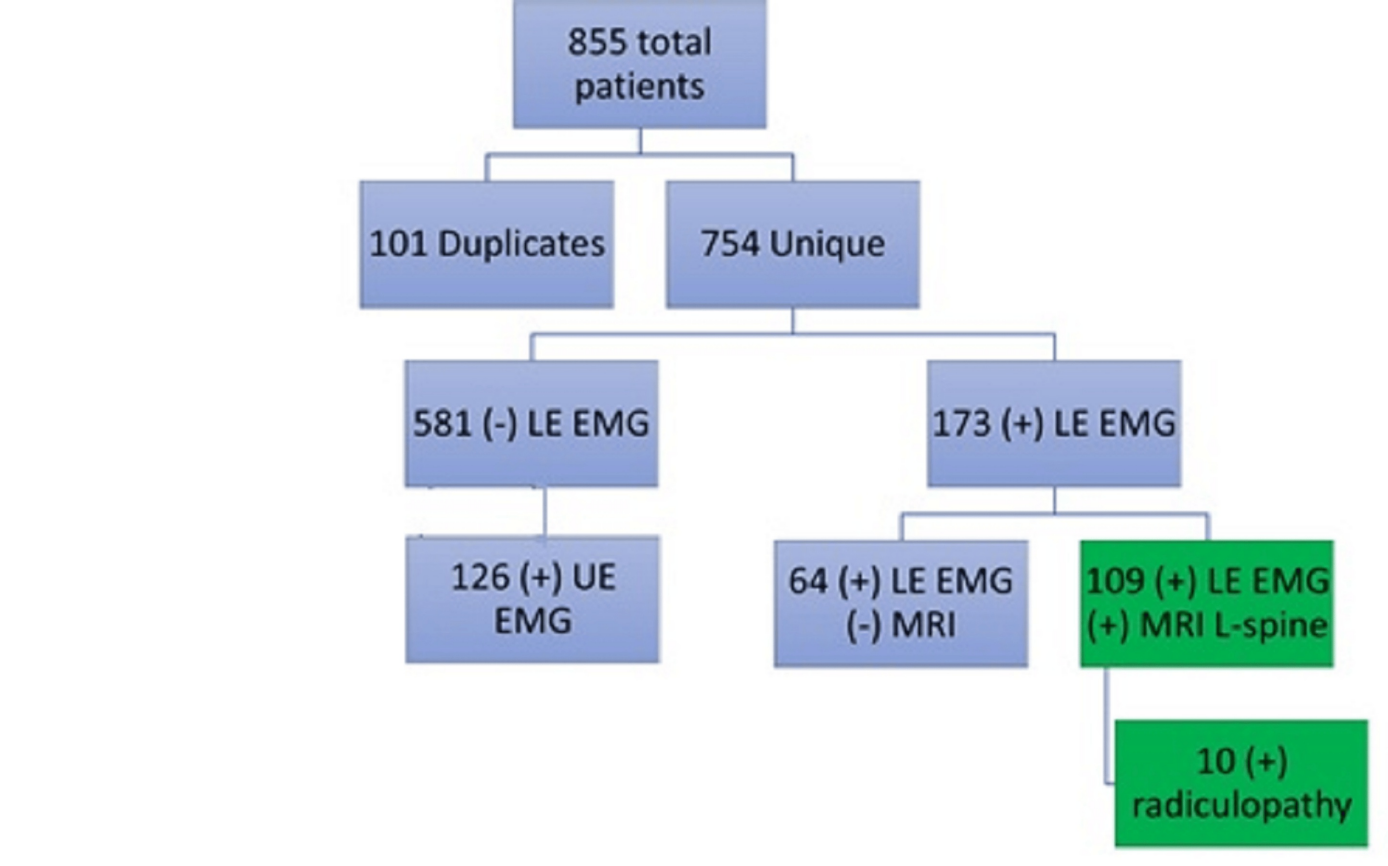
Patient selection algorithm Eight hundred and fifty-five total patients were identified based on chart review. 101 of these patients were identified to be duplicate records and were removed from the study, leaving 754 unique patients that were further reviewed. Of the 754 patients, 581 patients did undergo LE EMG at the time of the study, and 126 did undergo UE EMG. 173 patients did undergo LE EMG; however, 64 of the 173 patients did not have a coinciding lumbar MRI. This left 109 total subjects that had both MRI of the L-spine and LE EMG. 10 of these patients did have evidence of radiculopathy on EMG testing. LE: lower extremity; UE: upper extremity; EMG: electromyography; MRI: magnetic resonance imaging; L-spine: lumbar spine; (-): lack of testing; (+): presence of testing.

This retrospective cohort study was granted IRB approval through Wayne State University. Retrospective chart review helped to identify 855 potential patients. We started the inclusion criteria by considering all patients who had visited our outpatient pain medicine practice. We later identified the patients who had EDX and MRI available for review. Of these, 101 were duplicate patients. Of the remaining 754 unique patients, 126 individuals only had upper extremity EMG/nerve condition studies (NCS) testing results, and 455 individuals did not have a lower extremity EMG/NCS available for review. Sixty-four individuals had lower extremity NCS/EMG performed; however, they did not have any MRI studies available. The study pool was narrowed down to 109 total patients who had readily available lumbar MRI and complimentary EDX for review. Of the 109 research subjects, only 10 were identified to have EDX evidence of radiculopathy (Figure 1). All patients selected were coded and de-identified prior to statistical analysis. The EDX was performed within our neurology department, and all MRIs were read by neuroradiologists at our medical center.

Data Gathering

Two independent PM&R physicians were assigned to gather data in regards to EDX and lumbar MRI results in the 109 research subjects. For the EDX testing, we coded the EMG results for the 109 patients as either confirmed radiculopathy or absent and then assigned binary values. Routinely in the normal course of practice, images were independently reviewed by a board-certified neuroradiologist to determine the presence and severity of both LSS and LNS. Data were then separated into the presence or absence of LSS and the presence or absence of LNS based on the radiologist’s interpretation. Then, these subjects were further stratified into specific groups representing stenosis severity (none = 0, mild = 1, mild/moderate = 2, moderate = 3, moderate/severe = 4, severe = 5). The researcher assigned to the EDX results was blinded to the MRI results and vice versa.

Statistical analysis

We initially chose the chi-square test to examine the statistical association between radiculopathy and LSS, LSS severity, LNS, and LNS severity. We coded radiculopathy as present or absent, along with LSS and LNS severity. LSS severity and LNS severity were expressed as ordinal scale values ranging from 0 to 5. When the chi-square analysis was conducted, we found that the expected frequencies for many of the cells were below 5. Therefore, we analyzed the data using Fisher’s exact test and the Fisher-Freeman-Halton test.

## Results

The proportion of patients who had tested positive for radiculopathy on EDX testing was low (10/109, 9.17%) (Figure 1). Patients were further characterized and differentiated by the degree of LSS or LNS seen on MRI. Forty-two (38.5%) patients were determined to have some degree of LSS on imaging, while 86 (78.9%) patients were determined to have some degree of LNS on imaging. Within the LSS population, 24 (22.0%) patients had mild (grade 1), 2 (1.8%) patients had mild-moderate (grade 2), 8 (7.3%) patients had moderate (grade 3), no patients had moderate-severe (grade 4), and 8 (7.3%) patients had severe (grade 5) degrees LSS. Within the LNS population, 33 (30.3%) patients had mild (grade 1), 4 (3.7%) patients had mild-moderate (grade 2), 24 (22.0%) patients had moderate (grade 3), 5 (4.6%) patients had moderate-severe (grade 4), and 20 (18.3%) patients had severe (grade 5) degrees of LNS. Sixty-seven (61.5%) patients did not have any evidence of LSS on neuroimaging, and 23 (21.1%) of the patients did not have any evidence of LNS on neuroimaging (Table 1).

**Table 1:** Characterization of lumbar stenosis The 109 selected patients were further characterized based on degree of LSS and LNS. 42 (38.5%) total patients had evidence of LSS on MRI. Of these, 8 patients were characterized as severe (7.3%), 0 patients were moderate/severe, 8 patients were moderate (7.3%), 2 patients were mild/moderate (1.8%), and 24 patients were mild (22.0%) in terms of LSS severity. 86 (78.9%) patients had evidence of LNS on MRI. Of these, 20 patients were characterized as severe (18.3%), 5 patients were moderate/severe (4.6%), 24 patients were moderate (22.0%), 4 patients were mild/moderate (3.7%), and 33 patients were mild (30.3%) in terms of LNS severity. 67 (61.5%) patients had no evidence of LSS on MRI and 23 (21.1%) patients had no evidence of LNS on MRI; Severity Grading 0=None, 1=Mild, 2=Mild/moderate, 3=Moderate, 4=Moderate/severe, 5=Severe. LSS: lumbar spinal stenosis, LNS: lumbar neuroforaminal stenosis.

Severity grade	LSS	LSS%	LNS	LNS%
0	67	61.5	23	21.1
1	24	22.0	33	30.3
2	2	1.8	4	3.7
3	8	7.3	24	22.0
4	0	0	5	4.6
5	8	7.3	20	18.3
Total	42	38.5	86	78.9

Additionally, a multivariate logistic regression model was used to examine the set of variables (presence of lumbar stenosis, stenosis severity, presence of neuroforaminal narrowing, and severity of neuro-foraminal narrowing) to predict the presence of radiculopathy. There was no evidence of multicollinearity among the predictor variables; no VIF exceeded 2.60. Overall, the model was not statistically significant (p = 0.76), and none of the individual variables were significant predictors of radiculopathy. The c-statistic for this model was 0.61, which indicates poor model discrimination (Table 2).

**Table 2:** Summary of correlations FFH (Fisher-Freeman-Halton) testing was performed. There were no statistically significant associations between evidence of radiculopathy on EMG and the presence of LSS (p=0.50), LSS_sev (p=0.54), LNS (p=0.69), or LNS_sev (p=0.11). There were none to slight associations based on interrater reliability testing between evidence of radiculopathy on EMG and presence of LSS (k=0.052) and LNS (k=0.028). Variables of LSS, LNS, LSS_sev, and LNS_sev were fit to a multivariable logistic regression model to examine the potential to predict evidence of radiculopathy on EMG. The model was not statistically significant (p=0.76) and had poor model discrimination (c-statistic=0.61). LSS: lumbar spinal stenosis; LNS: lumbar neuroforaminal stenosis; LSS_sev: lumbar spinal stenosis severity; LNS_sev: lumbar neuroforaminal stenosis severity.

Fisher correlations (p-values)							
EMG result	LSS		LSS_sev		LNS		LNS_sev
Radiculopathy	0.50		0.54		0.69		0.11
Interrater reliability (k-values)							
Radiculopathy				0.052		0.028	
Multivariable logistic regression model							
p-value		c-statistic					
0.76		0.61					

## Discussion

The degree of LSS/LNS and associated findings on EDX had not been evaluated prior to this study. Specifically, no studies that we are aware of have assessed neuroforaminal narrowing either. The natural history of LSS with moderate symptom levels rarely shows symptom deterioration over a median of 3.3 years [20]. Moreover, it has been found that the probability of spontaneous activity on EDX is also not related to symptom duration [21]. This is unlike the known evolution of findings associated with acute nerve pathology and the emergence of neural changes apparent on EDX 3-6 weeks after insult.

Lumbar pain generalization can have several causes. The standard of care begins with conservative treatment options, including non-steroidal anti-inflammatories, physical therapy, chiropractic care, lifestyle modification and postural changes. Physical examination remains the best clinical indicator of geared diagnostic and treatment strategies.

Our data suggest that EDX may not be helpful in setting vague symptoms, especially without clear neurological findings on a physical exam. MRI and EDX can place additional burdens on the healthcare system and costs on patients, and ultimately, they may not have clinical significance. Clinically, most patients with LSS/LNS will improve with conservative treatments, including physical therapy, medications such as NSAIDs, and minimally invasive spinal interventions.

Survey data suggest that from 2017 to 2019, 10.8% of adults carried medical debt, including 10.5% of the privately insured and 9.6% of residents of Medicaid-expansion states, significantly fewer than in non-expansion states [22]. We have noticed an increase in utilization review systems employed by insurance companies and hospital systems.

MRI and EDX are not tolerated by all parties. Between 1% and 15% of all patients who undergo an MR examination suffer from claustrophobia and cannot be imaged, or they require sedation to complete the scan [23]. In terms of patient-centered care, clinicians must also remember that EDX testing is invasive, and the risks of adverse events, although relatively low, include pain/discomfort, infections, bleeding, lymphedema, pneumothorax, and many more [24,25].

There are several limitations to our study. Comparisons and statistical analyses were made on qualitative measures that were quantified. We did not observe positive associations between MRI results and electrodiagnostic evidence for radiculopathy, as previously reported [15]. Instead, we quantified MRI and EDX in a retrospective fashion. Interpretations of MRI were not standardized or controlled by one radiologist.

Other limitations include the lack of inclusion of physical examination findings, duration of symptoms, treatment options completed, or lumbar spine X-ray findings. We acknowledge a large pitfall is the absence of a correlation between diagnostic studies and physical examination findings.

## Conclusions

We observed no correlation between the degree of LSS/LNS on MRI and the EDX evidence of radiculopathy. Given the lack of correlation, EDX testing is not indicated prior to the trial of more advanced interventions. However, for individuals who fail to improve with expectant management, EMG testing and more detailed anatomical imaging are indicated.
